# Lipid signature in X-ALD: a comparison between phenotypes

**DOI:** 10.3389/fmolb.2026.1906499

**Published:** 2026-09-18

**Authors:** Alessandra Di Veroli, Sara Petrillo, Caterina Torda, Federica Loia, Teresa Rizza, Rosalba Carrozzo, Gabriele Cruciani, Enrico Bertini, Francesco Nicita, Fiorella Piemonte, Marco Cappa

**Affiliations:** 1 Department of Chemistry, Biology and Biotechnology, University of Perugia - C.U.R.I. Centro Umbro di Ricerca e Innovazione, Rome, Italy; 2 Unit of Muscular and Neurodegenerative Diseases, Neuromuscular Diseases Research Unit, Bambino Gesù Children’s Hospital, IRCCS, Rome, Italy; 3 Laboratory of Medical Genetics, Translational Cytogenomics Research Unit, Bambino Gesù Children’s Hospital, IRCCS, Rome, Italy; 4 Research Unit for Innovative Therapies in Endocrinopathies, Bambino Gesù Children’s Hospital, IRCCS, Rome, Italy

**Keywords:** AMN, CALD, lipid signature, lipidomics, X-ALD

## Abstract

**Background:**

X-linked adrenoleukodystrophy (X-ALD) has highly variable phenotypes with no known genotype/phenotype correlation or method for predicting the course of the disease. Screening for X-ALD allows for early, potentially lifesaving treatment of adrenal insufficiency and cerebral demyelination, possibly useful to detect pre-clinical differences in patients and extend treatments to pre-symptomatic subjects. In this study, we analyzed the lipid signature in fibroblasts of patients with Adrenomyeloneuropathy (AMN), the late onset and slowly progressive form of X-ALD, and in patients with Cerebral Adrenoleukodystrophy (CALD), the cerebral inflammatory demyelinating form of early childhood, in order to identify specific lipid molecules as potential early predictors for CALD.

**Methods:**

We analyzed lipidomic profiles in fibroblasts obtained from n = 4 clinically affected (and genetically proven) X-ALD patients (two CALD and two AMN) and n = 4 age-matched controls, using a Dionex UltiMate 3000 UHPLC system, coupled to a Q-Exactive mass spectrometer.

**Results:**

Our results highlight alterations in lipid metabolism both in AMN and in CALD fibroblasts relative to controls, further confirming the role of LPC 26:0 as a potential biomarker of X-ALD. However, the extent and pattern of lipid remodeling differ substantially between the two phenotypes, with a contribution of neutral lipids in CALD patients, along with a selective involvement of gangliosides.

**Conclusion:**

Although both AMN and CALD fibroblasts share some disease-associated lipid alterations, CALD samples exhibited a broader and more pronounced perturbation in multiple lipid classes, particularly in LPCs and steryl esters. The identification of phenotype-associated lipid signatures may represent a step forward toward precision medicine in X-ALD, in order to improve patient phenotypic evaluation.

## 1 Introduction

X-linked adrenoleukodystrophy (X-ALD) is a peroxisomal neurometabolic disorder caused by pathogenic variants in the ABCD1 gene, leading to dysfunction of the ABCD1 transporter required for β-oxidation of very long chain fatty acids (VLCFA) (Azar et al., 2020; Turk et al., 2020; Engelen et al., 2014; Engelen et al., 2012).

In recent years, several countries have introduced X-ALD in the newborn screening (NBS) programs, first implemented in New York in 2013 and later adopted across the United States. Pilot studies are ongoing in China, India, and the Netherlands (Eng and Regelmann, 2020). In Italy, NBS currently includes 49 conditions, but not X-ALD, although regional pilot study has started in 2021 in Lombardy and is ongoing in a few other regions (Bonaventura et al., 2023; Videbæk et al., 2023).

A major challenge in X-ALD is not only diagnosis but also the limitations of current screening strategies. Detection relies mainly on the VLCFA accumulation, with C26:0-lysophosphatidylcholines (LPC) as a primary biomarker and potential mediator of X-ALD (Hubbard et al., 2006; 2009; Huffnagel et al., 2017; Jaspers et al., 2020; 2026; Morales-Romero et al., 2024; Billington et al., 2025). However, predicting clinical disease phenotype remains currently unresolved, despite its importance for treatment decisions.

According to the age of onset, the neurological lesions, and the disease progression, the phenotypic presentation consists of progressive cerebral X-ALD (CALD), Addison’s only form that can evolve towards adrenomieloneuropathy (AMN) and asymptomatic or mild affected carriers (Engelen et al., 2012). About 40% of patients with pathogenic ABCD1 variants develop CALD during childhood with a rapidly progressing inflammatory demyelination (Turk et al., 2020; Azar et al., 2020). CALD is fatal if left untreated, and progression is usually rapid (Zhu et al., 2025).

Genetic analysis of the ABCD1 gene is widely recognized as a reliable and definitive method for diagnosis of the disease, including both targeted sequencing and the Next-Generation Sequencing (NGS) or whole-exome sequencing approaches (Zhu et al., 2025). More than 1040 gene variants have been described (The ABCD1 Variant Database, 2022), but no correlation between genotype and phenotype has been found (Eng and Regelmann, 2020). Indeed, the same ABCD1 mutation may lead to CALD, AMN, or Addison’s disease in different individuals (Ozdemir Kutbay et al., 2019) but also in genetically identical twins (Korenke et al., 1996; Di Rocco et al., 2001), thus proving that genetic background is not the only determinant of the phenotypic heterogeneity in this disease.

VLCFA accumulation, particularly C26:0-lysophosphatidylcholine (LPC(26:0)), is the primary biochemical marker of X-ALD (Marten et al., 2026; Hein et al., 2008; Kruska et al., 2015; Jaspers et al., 2026). LPC(26:0) arises from the accumulation of VLCFA-CoA species that are incorporated into phosphatidylcholine via the lysophospholipid acyltransferase LPLAT10, followed by phospholipase A2-mediated hydrolysis (Jaspers et al., 2026). LPC(26:0) accumulation in CNS lipoproteins would be responsible for neurotoxic and proinflammatory effects observed in experimental models and recent evidence suggest a correlation among high levels of LPC(26:0), severe spinal cord disease and cerebral ALD (Eichler et al., 2008; Carneiro et al., 2013; Prabutzki et al., 2024; Fujitani et al., 2024; Jaspers et al., 2026). The Neurofilament light chain content has also been proposed as a prognostic biomarker for early detection of CALD, in addition to current neuroimaging predictors (Weinhofer et al., 2021; 2023; Wang et al., 2022). However, to date, biomarkers-based risk profiles able predicting CALD are still lacking, despite several multi-omics analyses have been carried out (Richmond et al., 2020; Mallack et al., 2022; Honey et al., 2021). The extent of VLCFA overload itself does not correlate with the severity of the disease and cannot fully explain the clinical phenotypes of patients (Turk et al., 2020).

In this study, we applied an untargeted lipidomic approach by liquid chromatography-mass spectrometry (LC-MS/MS) to analyze lipid profiles of skin fibroblasts obtained from n = 2 patients with AMN, n = 2 with CALD, and n = 4 age-matched healthy subjects. The aim was to identify a lipid signature potentially useful for early prediction of pre-symptomatic subjects and possibly define risk-profiles for the development of the cerebral form of the disease. Our findings, despite the limited sample size, nevertheless may provide a basis for identifying lipid predictors in CALD and support future studies for early patient stratification.

## 2 Materials and methods

### 2.1 Fibroblasts cultures

Skin biopsies were taken from n = 4 clinically affected (and genetically proven) X-ALD patients (two CALD and two AMN) and n = 4 age-matched controls (Ctrls). Patient biopsies were performed at diagnosis, and patients were not taking any medication or following a special diet. Fibroblasts were grown in Dulbecco’s modified Eagle’s medium supplemented with 10% fetal bovine serum, 50 units/mL penicillin, 50 ug/mL streptomycin, 0.4% (v/v) amphotericin B (250 ug/mL), at 37 °C in 5% CO2. Cells were used at similar 9–11 passage numbers, tested to exclude *mycoplasma* contamination, and cultured for approximately 20 days before lipid extraction. The assays were performed in triplicates and all participants have been signed an informed consent. Each sample consisted of 1 × 10^6^ cells. The study was conducted in accordance with the Declaration of Helsinki, and approved by the OPBG Ethics Committee (2058, 1 June 2018).

### 2.2 Lipid extraction and LC–MS/MS analysis

Lipid extraction in fibroblasts was performed with the MMC mixture (Methanol:MTBE:chloroform, 40:30:30, v:v:v) which enables comprehensive untargeted lipidomic profiling (Gianni et al., 2022; Pellegrino et al., 2014). The MMC (Methanol:MTBE:chloroform) extraction protocol was chosen for untargeted lipidomics due to its broad lipid coverage and reduced sample manipulation. Its monophasic nature minimizes potential lipid losses during phase separation, representing an advantage when analyzing limited amounts of patient-derived cellular material. Briefly, an opportune volume of the cold solvent mixture (methanol:MTBE:chloroform, 40:30:30, v/v/v), supplemented with butylated hydroxytoluene (BHT, 100 mg/L) as an antioxidant, was added to each sample spiked with the EquiSPLASH™ LIPIDOMIX™ (Avanti Polar Lipids Inc., Alabaster, AL, USA) containing the following lipids: (15:0-18:1 (d7) PC, 18:1 (d7) Lyso PC, 15:0-18:1 (d7) PE, 18:1 (d7) Lyso PE, 15:0-18:1 (d7) PG, 15:0-18:1 (d7) PI, 15:0-18:1 (d7) PS, 15:0-18:1 (d7)-15:0 TAG, 15:0-18:1 (d7) DAG, 18:1 (d7) MAG, 18:1 (d7) Chol Ester, d18:1-18:1 (d9) SM, C15 Ceramide-d7) at the concentration of 2 μg/ml and were used for multi-standard normalization of signals that were normalized using a two-step approach. First, lipid abundances were normalized according to the number of cells used for each extraction to account for differences in biological input. Subsequently, multi-standard normalization was performed using the EquiSPLASH™ internal standard mixture. Because lipid identification was performed using both positive and negative electrospray ionization modes, normalization was performed considering ionization polarity and adduct formation.

After adding solvents, samples were vortex-mixed and incubated for 30 min at room temperature in a thermomixer under agitation (950rpm), followed by centrifugation for 10 min at room temperature (8000 rpm). The resulting supernatants were collected, and a 5 µL aliquot was injected for LC–MS/MS analysis.

All samples were analyzed using a Q-Exactive Orbitrap mass spectrometer from Thermo Fisher Scientific coupled to a Dionex Ultimate 3000 high-performance liquid chromatography system.

Lipid separation was carried out on a Thermo Accucore™ C18 HPLC column (150 mm × 2.1 mm, 2.6 µm particle size) using gradient elution. Mobile phase A consisted of MeCN/H_2_O (1:1, v/v), while mobile phase B was i-PrOH/MeCN/H_2_O (85:10:5, v/v/v); both solvents contained 5 mM ammonium formate and 0.1% (v/v) formic acid. Chromatography was performed at 50 °C with a flow rate of 0.3 mL/min under the following gradient program: 0–20 min, 10%–86% B (curve 4); 20–22 min, 86%–95% B (curve 5); 22–26 min, 95% B isocratic; 26–26.1 min, 95%–10% B (curve 5), followed by 5 min re-equilibration at 10% B.

Samples were initially acquired in Full MS mode with positive/negative ion polarity switching. Subsequently, a subset of injections was analyzed in DDA positive and DDA negative modes to obtain MS/MS spectra. Electrospray ionization settings were: sheath gas 40 a.u., auxiliary gas 10 L/min, sweep gas 1 L/min, spray voltage 3.5 kV (positive mode) and 2.5 kV (negative mode), ion transfer temperature 300 °C, S-lens RF level 35%, and auxiliary gas heater temperature 370 °C.

LC–MS/MS datasets from each biological matrix were processed and annotated using Lipostar2 (Mass Analytica, version 2.2.1) (Goracci et al., 2017). The software was used for raw data management, peak detection, feature alignment, blank subtraction, lipid identification and multivariate statistical analysis (PCA and OPLS-DA). Lipid species were characterized in both positive and negative ionization modes by comparing experimental MS/MS spectra with the Lipostar reference library and by applying class-specific fragmentation rules. Raw files were imported and aligned using MS intensity thresholds of 20,000 in positive mode and 10,000 in negative mode, together with an MS/MS filtering threshold of 0.99. Peak smoothing was performed using the SDA algorithm set to “low,” and peak detection employed an m/z tolerance of 2 ppm. Isotopic clustering used a mass tolerance of 0.01 amu and a retention time (RT) window of 0.2 min. For sample alignment, tolerances of 0.02 (mass) and 0.5 min (RT) were applied. An MS/MS-based filter retained only features supported by fragmentation spectra. Lipid annotations were accepted when they achieved a quality score of 3–4 stars, corresponding to the highest confidence levels; these assignments were then visually inspected and further refined according to peak quality and MS/MS spectral consistency.

### 2.3 Statistical analysis

Two independent sample sets were analyzed. The first dataset consisted of fibroblasts from n = 2 adult patients affected by AMN and n = 2 adult controls, while the second dataset included fibroblasts from n = 2 pediatric patients with CALD and n = 2 pediatric controls.

Statistical analyses were performed using LipoStar2 (Mass Analytica, version 2.2.1) and GraphPad Prism (version 10.4.2). Data were Pareto scaled prior to multivariate analysis. Principal component analysis (PCA) was applied to assess data quality, detect outliers, and evaluate intrinsic sample clustering. Orthogonal partial least squares–discriminant analysis (OPLS-DA) was subsequently performed to facilitate the identification and interpretation of lipids contributing to group separation, which was already observed in the unsupervised PCA. Variables with a variable importance in projection (VIP) score >1 were considered relevant for discrimination. Model robustness was assessed by internal leave-one-out cross-validation (LOOCV). Given the limited sample size, both the PCA and OPLS-DA models should be considered exploratory within the context of this pilot study.

Univariate analysis was conducted using unpaired t-tests with Welch’s correction. P-values were adjusted for multiple comparisons using the Benjamini–Hochberg procedure, and corrected p-values (corr p-values) <0.05 were considered statistically significant. Fold change (FC) analysis was also performed, and lipids with FC > 2 were considered biologically meaningful. Results were visualized using volcano plots.

A set of robust lipid markers was defined by selecting features meeting all the following criteria: VIP >1, corr p-value <0.05, and FC > 2. Distribution of selected lipids across groups was visualized using violin plots, with statistical significance assessed as described above.

## 3 Results

For both datasets, the first consisting of n = 2 AMN fibroblasts and n = 2 adult controls, while the second included n = 2 CALD fibroblasts and n = 2 pediatric controls, the same untargeted lipidomics approach was applied. The aim was to characterize the lipidomic fingerprint of these two X-ALD phenotypes, identifying differences relative to controls, and exploring potential lipidomic differences between the AMN and CALD forms as a basis for the identification of early biomarkers.

The use of fibroblasts instead of plasma allowed the analysis of a biological matrix less influenced by diet, pharmacological treatments, or dietary supplements. Furthermore, fibroblasts are a good cell model for X-ALD, recapitulating the main disease hallmarks (López-Erauskin et al., 2012; López-Erauskin et al., 2013; Launay, 2015, Launay, 2017), and represent an inexhaustible source of information, due to their long-term storage.

Analysis of the lipidome of the first dataset resulted in the identification of approximately 600 lipid species belonging to multiple classes. Glycerophospholipids represented the most abundant lipid category, with diacylglycerophosphocholines (PC, n = 132), followed by ether-linked PCs (ether-PC, n = 72) and phosphatidylethanolamines (PE, n = 59). Other glycerophospholipid classes (PI n = 39, LBPA n = 33, ether-PE n = 28, PS n = 28), as well as sphingolipids (gangliosides n = 36, ceramides (Cer) n = 25, sphingomyelins (SM) n = 18), were also well represented. The relative composition of the lipidomic fingerprint is reported in Figure 1A.

**FIGURE 1:**
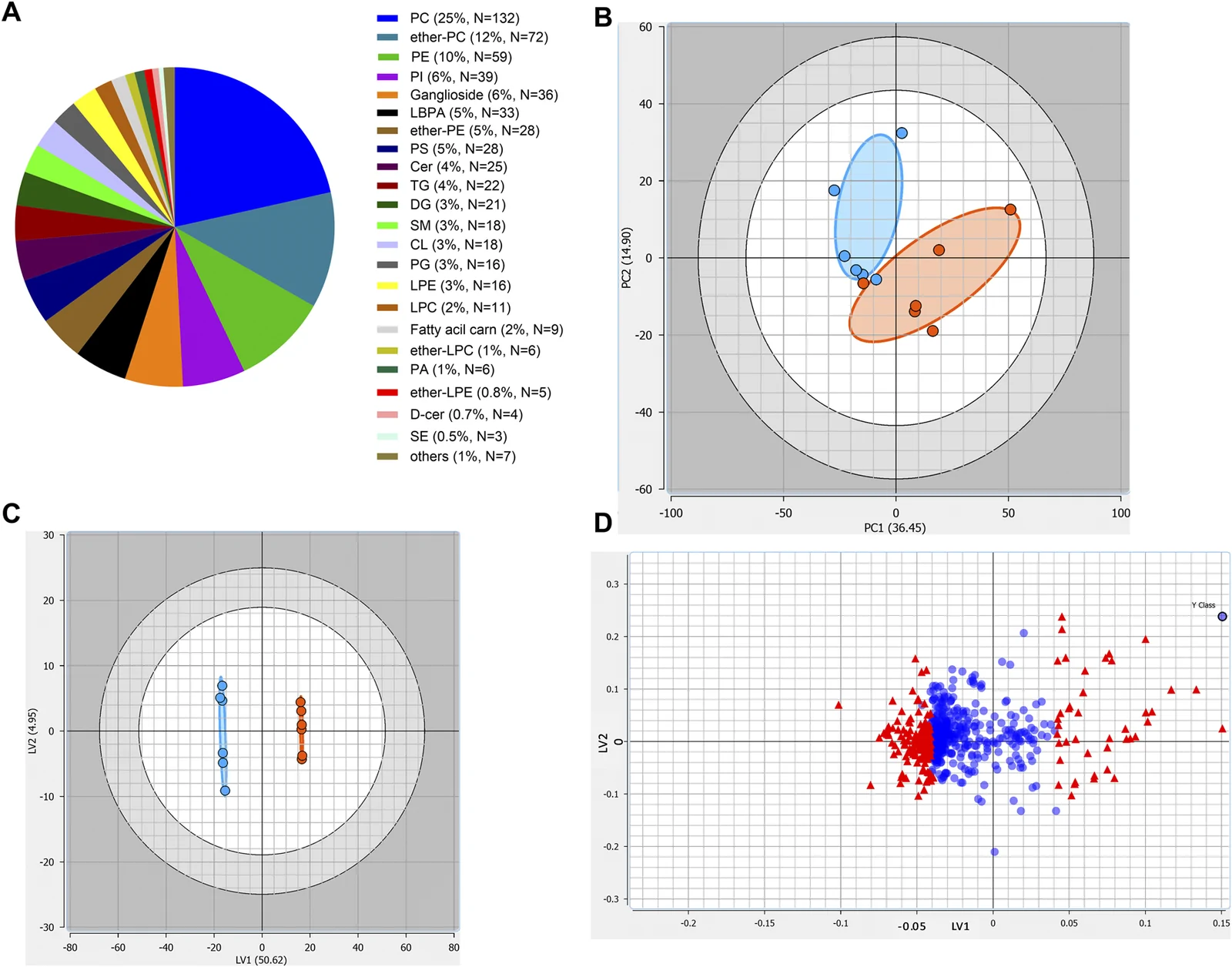
Lipidomic fingerprint and multivariate analysis of AMN fibroblasts. Comprehensive characterization of the lipidome discrimination of n = 2 patient-derived cells. Experiments were performed in biologicals triplicates for controls and patients. **(A)** The pie chart represents the percentage and the total number (N) of unique lipid species identified within each class. Phosphatidylcholines (PC, 25%) and ether-Phosphatidylcholines (ether-PC, 12%) constitute the most represented lipid families. **(B)** Principal Component Analysis (PCA) score plot illustrating the unsupervised separation between AMN patients (orange) and healthy controls (blue) based on the global lipidomic profile. **(C)** OPLS-DA score plot showing the supervised discrimination between the two experimental groups along the predictive component (LV1). **(D)** OPLS-DA loading plot showing the distribution of individual lipid species. Red triangles indicate high-impact variables (VIP> 1).

To investigate global lipidomic variation, an unsupervised principal component analysis (PCA) was performed (Figure 1B). The score plot revealed a distinct clustering of AMN fibroblasts compared to healthy controls along the first principal component (PC1) despite some overlap, indicating a significant shift in the overall lipid metabolism of patient-derived cells.

To further maximize the separation between AMN and control groups and identify the most discriminating lipid species, a supervised OPLS-DA model was performed, and variables with a Variable Importance in Projection (VIP) score greater than one were selected. In Figures 1C,D, the OPLS-DA t/t score plot and the loading plot are respectively showed.

The OPLS-DA score plot (Figure 1C) demonstrated a clear and robust separation between the two cohorts along the predictive component (LV1), which accounted for 51% of the total variance. In the corresponding loading plot (Figure 1D) the high-impact (VIP >1) lipids are highlighted with red triangles and represent the variables that drive the metabolic divergence observed in patient-derived fibroblast, highlighting the rearrangement of the lipidome in the AMN phenotype. In this group of about 200 lipids, diacylglyerophosphocholines (PC) were the most abundant with 32 species, followed by their ether forms (ether-PC, n = 26). Similar abundance showed other glycerophosholipids (PE n = 16, ether-PE n = 14, PI n = 11) and sphingolipids classes (Cer n = 14, gangliosides n = 12, SM n = 12).

In parallel, univariate analysis was conducted to evaluate the magnitude and statistical significance of lipid changes (Figure 2). Lipids with an absolute fold change greater than two and a p-value <0.05 were considered significantly altered. The integration of multivariate and univariate approaches identified a subset of lipids fulfilling all selection criteria. These features highlighted in the volcano plot, although referring to only two patients, support biologically relevant differences between patients and controls, useful for further investigations on a larger cohort of patients.

**FIGURE 2:**
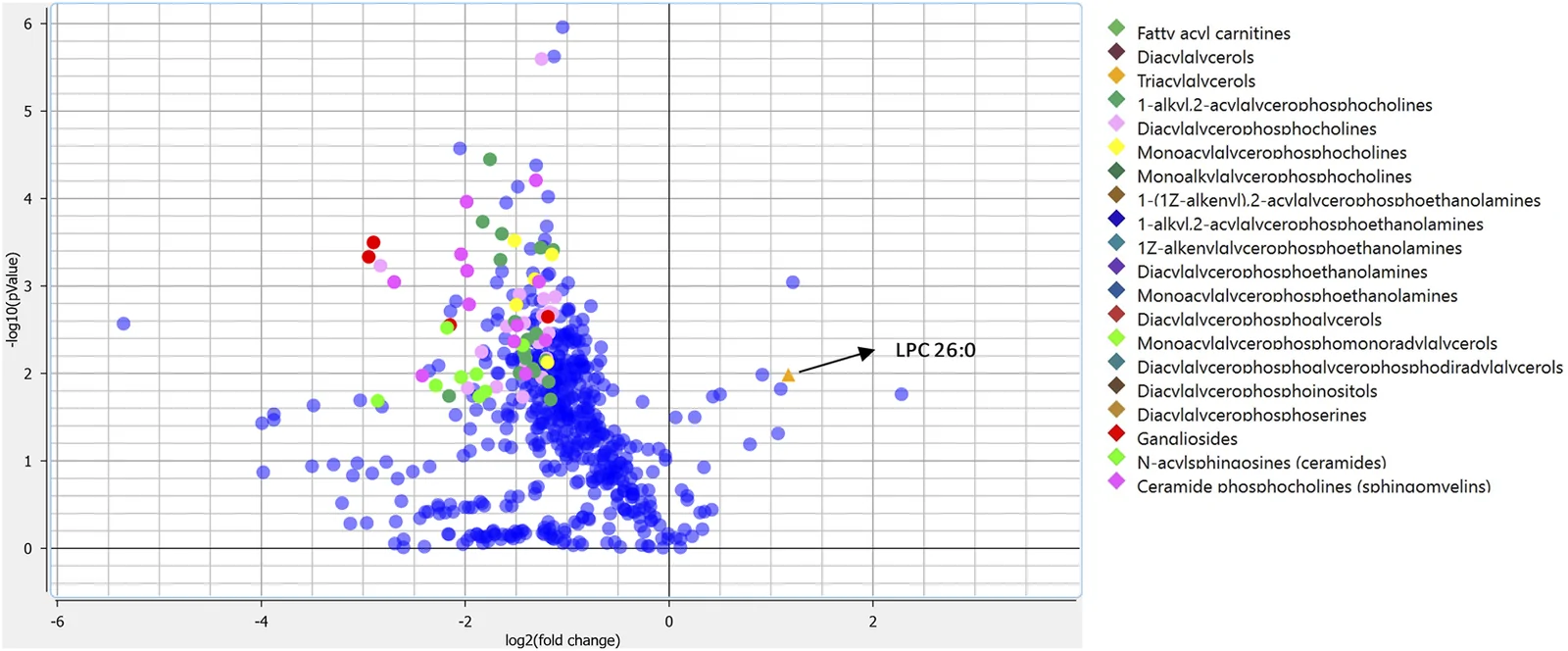
Quantitative distribution of high-impact lipid species identified by univariate analysis in AMN fibroblasts. The volcano plot displays the differential expression of individual lipid species within each class characterized by a Variable Importance in Projection (VIP) score >1, absolute Fold Change (FC) > 2 and corr p-value <0.05 (determined via Student’s t-test). Species are color-coded by lipid class according to the legend.

Interestingly, the global lipidomic profile of AMN fibroblasts was characterized by a predominant downregulation, as evidenced by the high density of significant lipid species on the left side of the plot (negative log2 fold change). Specifically, several classes, including the PC class (n = 20) and their ether-linked counterparts’ ether-PC (n = 18), followed by PE (n = 11) and ether-PE (n = 11), showed a marked decrease in AMN patients compared to controls. Sphingolipids were also represented, including SM (n = 12), Cer (n = 8) and gangliosides (n = 4). Conversely, only a limited number of lipid species exhibited a significant increase, suggesting a profound depletion or impaired synthesis of key structural and signaling lipids in the AMN phenotype.

Given the established role of LPC 26:0 as a plasma biomarker in X-ALD, particular attention was paid to the monoacylglycerophosphocholine (LPC) class, despite its relatively limited representation within the overall lipidome.

Among the 11 identified LPC species, seven showed VIP scores >1, indicating a relevant contribution to the group discrimination. Of these, two species (LPC 22:0 and LPC 26:3) contained long acyl chains, whereas the remaining species were characterized by shorter chains (≤C20), and all these lipids were significantly decreased in patients. LPC 26:0, although not meeting the VIP >1 threshold (VIP = 0.95), was retained for further consideration due to its known biological relevance. Notably, LPC 26:0 was among the few lipids found to be increased in patients with AMN compared to controls, as highlighted in the volcano plot (orange triangle, Figure 2).

The second dataset consisted of fibroblast samples obtained from two pediatric patients affected by cerebral adrenoleukodystrophy (CALD) and two age-matched pediatric controls. A total of approximately 1300 lipid species were identified.

The most abundant lipid classes in the lipidomic fingerprint were phosphatidylcholines (PC, n = 412) and phosphatidylethanolamines (PE, n = 146), followed by triglycerides (TG, n = 129), and ether-linked forms of PC and PE (ether-PC, n = 108; ether-PE, n = 92). Other phospholipid classes, including phosphatidylserines (PS) and phosphatidylinositols (PI), as well as diacylglycerols (DG), were also well represented. Sphingolipids were mainly represented by gangliosides (n = 38), sphingomyelins (SM, n = 40), and ceramides (Cer, n = 13). The relative composition of the lipidomic fingerprint is shown in Figure 3A.

**FIGURE 3:**
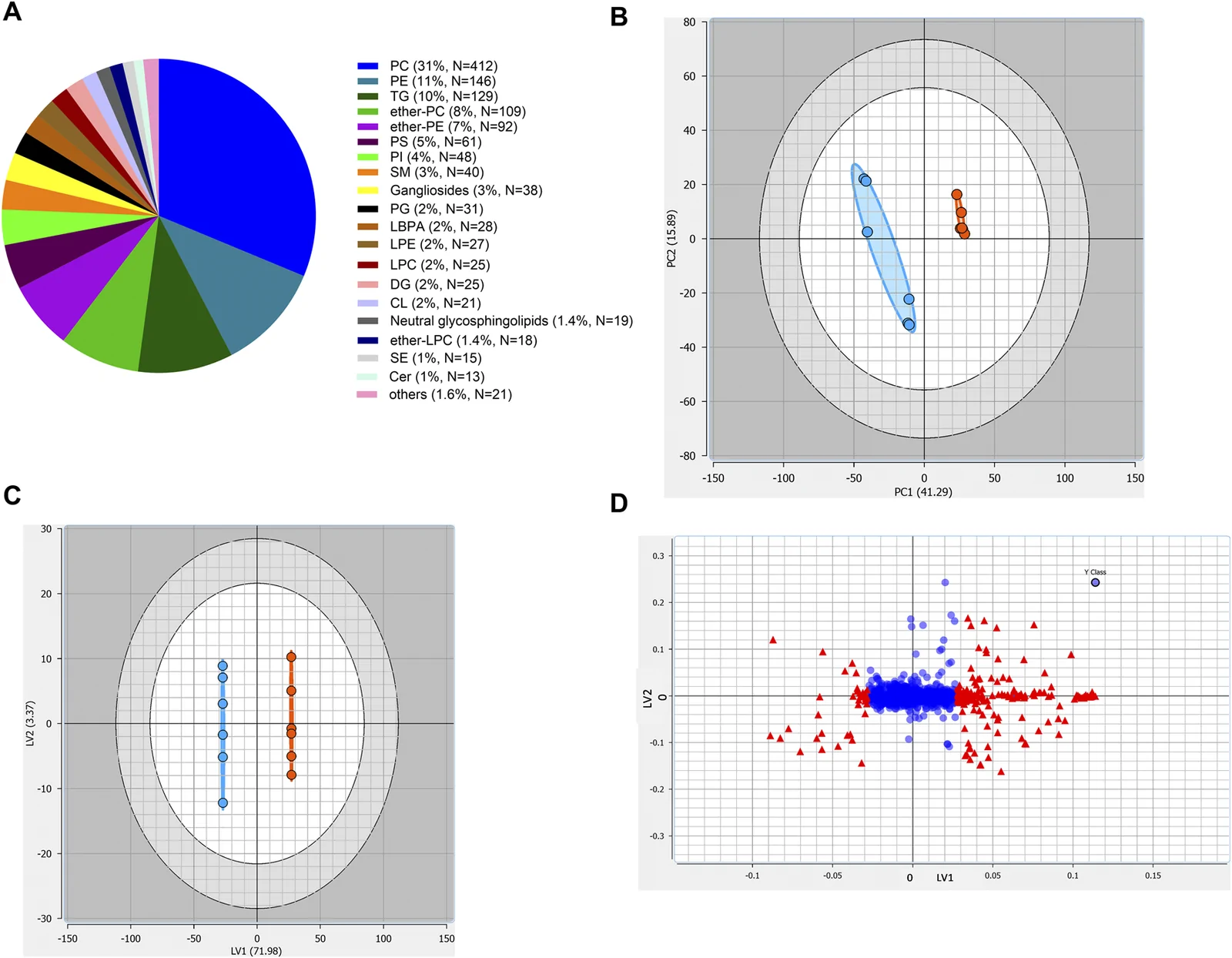
Lipidomic fingerprint and multivariate analysis of CALD fibroblasts. Comprehensive characterization of the lipidome discrimination in patient-derived samples from the cerebral adrenoleukodystrophy (CALD) cohort. Experiments were performed in biologicals triplicates for controls and patients. **(A)** Pie chart representing the percentage and the total number (N) of unique lipid species identified within each class. Phosphatidylcholines (PC, 31%) and Phosphatidylethanolamines (PE, 11%) represent the most abundant families identified. **(B)** Principal Component Analysis (PCA) score plot showing a clear unsupervised segregation between CALD patients (orange) and healthy controls (blue). **(C)** OPLS-DA score plot illustrating a robust supervised descrimination between groups along the predictive component (LV1). **(D)** Loading plot highlighting the individual lipid species contributing to the separation. Red triangles represent high-impact variables (VIP>1).

Principal component analysis (PCA) did not reveal the presence of outliers and showed a clear separation between patients and controls along PC1 (41.29% explained variance), indicating marked differences in lipidomic profiles (Figure 3B).

To investigate the lipidomic alterations specifically associated with the childhood cerebral form of the disease (CALD), a supervised OPLS-DA model was generated (Figures 3C,D). The score plot (Figure 3C) exhibited a striking and complete separation between CALD patient-derived fibroblasts and healthy controls along the predictive component (LV1), which explained a substantial portion of the total variance (71.98%). The robustness of this model was confirmed by the loading plot (Figure 3D), where several lipid species (highlighted as red triangles) emerged as key drivers of the clinical phenotype.

Lipids contributing to this discrimination were selected based on a VIP score >1 on the predictive component (LV1), resulting in a total of 224 lipid species. Among these, diacylglycerophosphocholines (PC) were the most represented class (n = 98), indicating a major contribution of this lipid class to the group discrimination. Notably, PCs with longer acyl chains and a higher degree of unsaturation tended to be more abundant in patients. Triglycerides (TG) represented the second most abundant class among VIP-selected lipids (n = 27). They were all increased in patients and, interestingly, all these species showed a total carbon number greater than 54, suggesting a shift toward longer-chain neutral lipids. Moderate representation was observed for phosphatidylethanolamines (PE, n = 20) and phosphatidylinositols (PI, n = 12), along with other lipid classes including gangliosides (n = 9), ether-PC (n = 10), and monoacylglycerophosphocholines (LPC, n = 4). Regarding sphingolipids, only a subset of gangliosides (9 out of 38 identified species) contributed to the group discrimination, and all were increased in CALD patients. In contrast, none of the identified ceramides or sphingomyelins showed relevant contribution to the model. While almost all identified steryl esters (SE) exhibited VIP >1 and were consistently increased in patients.

The quantitative univariate analysis of the CALD lipidome, visualized via volcano plot revealed a widespread and significant metabolic dysregulation. The intersection between univariate results (fold change >2, p-value <0.05) and VIP-selected variables yielded a subset of 140 lipids, highlighted in Figure 4. Within this subset of 140 lipids, phosphatidylcholines (PC) represented the most abundant class (n = 55), followed by triglycerides (TG, n = 27). Additional lipid classes, including phosphatidylethanolamines (PE, n = 12), phosphatidylinositols (PI, n = 9), were also represented. Notably, the majority of gangliosides (7 out of 9) and steryl esters (11 out of 15) fulfilling the selection criteria were significantly increased in patients compared to controls (up to log2 fold change >6).

**FIGURE 4:**
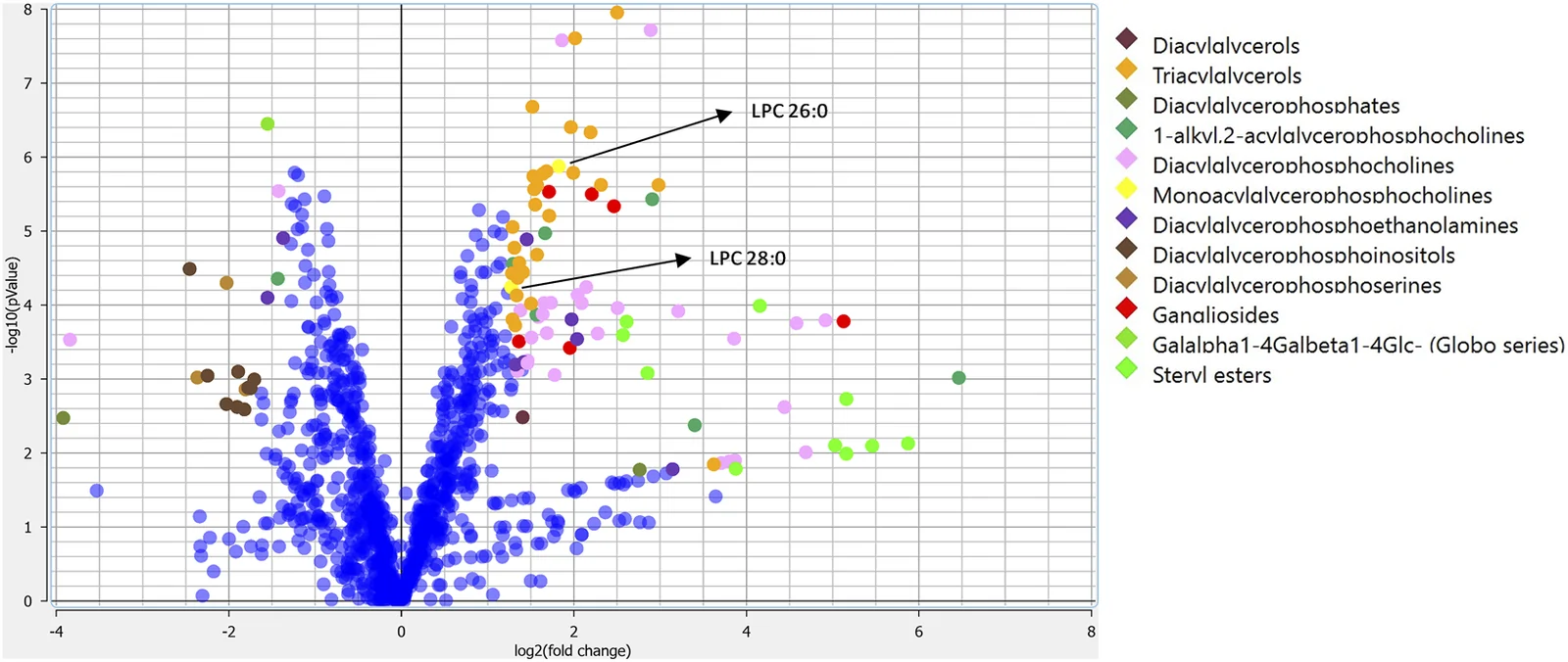
Quantitative distribution of high-impact lipid species identified by multivariate analysis in CALD fibroblast. The volcano plot displays the differential expression of individual lipid species within each class characterized by a Variable Importance in Projection (VIP) score >1, absolute Fold Change (FC) > 2 and corr p-value <0.05 (determined via Student’s t-test). Species are color-coded by lipid class according to the legend.

As in the previous dataset, particular attention was given to the monoacylglycerophosphocholines (LPC) class due to its known relevance in X-ALD. Among the 25 LPC species identified, only five showed VIP >1. Notably, LPC 26:0 was significantly increased in CALD-derived fibroblasts compared to controls. All selected LPC species were increased in patients. The integration of multivariate and univariate analyses within the LPC class revealed that only two species, LPC 26:0 and LPC 28:0, satisfied all selection criteria, further supporting their potential relevance as disease-associated lipid markers (Figure 5).

**FIGURE 5:**
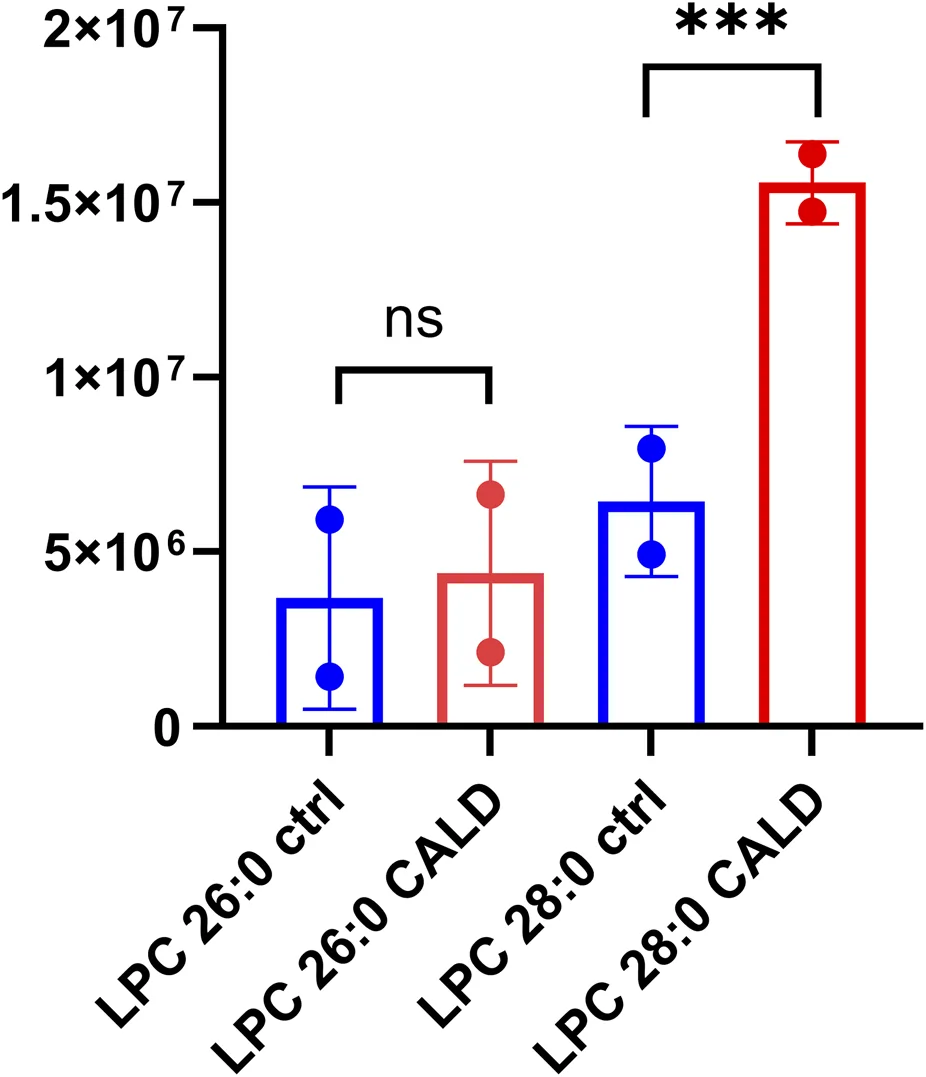
VLC-LPCs analysis in CALD fibroblasts. Violin plots showing the relative abundance of LPC 26:0 and LPC 28:0 in fibroblasts derived from healthy controls (blue) and patients with CALD (orange). Experiments were performed in biologicals triplicates for controls and patients. Each dot represents an individual biological replicate. Statistical significance was determined using Student’s t-test. ****p < 0.0001.

For both datasets the OPLS-DA models were primarily employed to facilitate the interpretation and identification of the lipid species contributing to the discrimination between the two groups, which were already clearly separated in the corresponding unsupervised PCA analyses. Model performance was internally validated using leave-one-out cross-validation (LOOCV). Both models showed excellent goodness of fit (R^2^ = 1.000) and good to excellent predictive ability (Q^2^ = 0.576 and 0.986, in AMN and CALD model respectively), supporting the robustness of the observed class separation. Model diagnostics were additionally inspected to verify the absence of influential outliers and to confirm the internal consistency of the models. Nevertheless, given the limited sample size, both the PCA and OPLS-DA analyses should be regarded as exploratory within the context of this pilot study.

A comparative evaluation of the two datasets highlighted both shared and distinct lipidomic features between AMN and CALD fibroblasts. The integration of multivariate and univariate analyses consistently identified a subset of robust discriminant lipids. In the AMN dataset, the majority of significant lipid species, including PCs, ether-PCs, and sphingolipids, were decreased in patients compared to controls. In contrast, the CALD dataset was characterized by a general increase in lipid species, particularly involving PCs, TGs, SE and gangliosides.

In particular, within sphingolipid classes, only gangliosides contributed to group discrimination and increased in CALD patients, whereas a broader representation of sphingolipids was observed among altered lipids in AMN with gangliosides together with ceramides and sphingomyelins.

Furthermore, in both datasets, LPC 26:0 was found to be increased in patients compared to controls, confirming its consistent association with the disease phenotype, but significant differences were observed for the other LPC species as they were all decreased in AMN patients, while in the CALD dataset the LPC 26:0 was increased together with another long chain species, the LPC 28:0.

Overall these results, although on a limited cohort of patients, nevertheless indicate that, both AMN and CALD fibroblasts exhibit alterations in lipid metabolism relative to controls, confirming the role of LPC 26:0 as biomarker of X-ALD. However, the extent and pattern of lipid remodeling differ substantially between the two phenotypes, with a contribution of neutral lipids in fibroblasts of CALD patients together with a selective contribution of gangliosides.

## 4 Discussion

Lipid abnormalities have long been reported in X-ALD, including accumulation of VLCFAs in complex lipids, such as glycerophosphocholines, cholesterol esters, and triglycerides (Igarashi et al., 1976; Theda et al., 1992; Wilson and Sargent, 1993; Kemp et al., 2005; Berger et al., 2014; Lee et al., 2019; Ferrer et al., 2025). These changes may alter membrane structure and function, contributing to the disease progression and evolution.

Our data show a general enrichment of VLCFAs across multiple lipid classes in fibroblasts obtained from n = 2 AMN and n = 2 CALD patients. Although fibroblasts are not representative of the most affected cells in X-ALD (such as oligodendrocytes and astrocytes), nevertheless they are an easily accessible and inexhaustible source of cells, not influenced by diet, pharmacological treatments, or dietary supplements, and a good disease model fully recapitulating the main X-ALD hallmarks (VLCFA accumulation, free radicals overload, energetic impairment).

Increased lipid species in X-ALD fibroblasts have been previously reported by Jaspers R et al. (2024), who identified LPC 26:0 as the most prominent biomarker, along with elevations in PC, TG, SM, and CE. It is worth noting that several earlier studies quantified VLCFAs mainly as total fatty acids after hydrolysis, regardless of the lipid species in which they were esterified. This approach provides an estimate of the overall VLCFA burden, but inevitably leads to a loss of structural information about lipids were these fatty acids are incorporated. Conversely, lipidomic approaches that preserve lipid species identity allow a more detailed characterization of the metabolic pathways affected by the disease. In this context, the work by Jaspers Y et al. (2024) is consistent with previous literature, showing an enrichment of lipids containing VLCFA in X-ALD samples compared with controls and evidencing LPC, PC, DG, TG, and CE as the main lipid classes involved. Notably, these lipid classes emerged also in our dataset as those most affected by disease-related lipid remodeling. However unlike Jaspers Y et al. (2024), who analyzed mixed phenotypes, we examined AMN and CALD separately.

In our dataset, sphingolipids strongly contributed to the AMN signature, while only gangliosides were relevant in CALD. Given their role in membrane organization and signaling, this selective involvement may reflect chronic membrane alterations in AMN. Conversely, triglycerides were increased mainly in CALD, suggesting enhanced lipid storage in the more severe phenotype.

Interestingly, redox imbalance may also contribute to the observed lipid remodeling. In a previous study from our group (Petrillo et al., 2022), we reported a marked redox impairment in X-ALD fibroblasts, with significantly higher levels of lipid peroxidation in CALD compared with AMN samples. These findings suggest that oxidative stress could represent an additional driver of lipid alterations in X-ALD and highlight the potential relevance of redox-lipidomics approaches for further investigating disease mechanisms.

A role for VLCFA incorporation into membrane phospholipids was previously proposed by Theda et al. (1992) in postmortem white matter from a patient with late onset X-ALD. They showed that VLCFA enrichment in phosphatidylcholines preceded demyelination, while VLCFA-containing cholesterol esters accumulated only in demyelinated areas. Our finding of increased long-chain steryl esters and triglycerides in CALD supports the activation of storage pathways as a compensatory response to lipid excess in this more aggressive phenotype, beyond the general remodeling of membrane lipids. Regarding LPC species, LPC 26:0 was elevated in both phenotypes, confirming its diagnostic value. However, LPC 28:0 was detected only in CALD, thus suggesting its potential specificity for the cerebral form and a possible complementary role to LPC 26:0. Overall, AMN and CALD share lipid remodeling in fibroblasts of our patients but differ in pattern. AMN shows broader sphingolipid involvement, whereas CALD is characterized by increased neutral lipids and storage pathways. These differences might reflect distinct metabolic responses, rather than simple VLCFA accumulation. From a biomarker perspective, sphingolipid alterations may characterize AMN, while increased triglycerides, steryl esters, and LPC 28:0 may define CALD.

From a mechanistic point of view, what is currently known on the relationship between lipids changes and disease progression in X-ALD is that the altered lipid composition, caused by the Abcd1-deficiency, can induce cells (microglia and astrocytes) to enter a reactive state, with secretion of neuro-toxic mediators, increase of cell susceptibility to ferroptosis and neuro-inflammation, ultimately leading to neurodegeneration and accelerating the disease progression (Fujitani et al., 2024; Tawbeh et al., 2025; Yu et al., 2022; Sahu et al., 2026; Salas et al., 2026; Tiwari and Simons, 2025; Marten et al., 2026; Petrillo et al., 2022; Kloss et al., 2026). In particular, increased VLCFA incorporation into myelin lipids may alter its structural compactness, providing a mechanistic link between VLCFA-containing lipid accumulation and early white matter pathology (Yska et al., 2025).

It is important to emphasize that, for the limited cohort of patients, this study should be considered rather a pilot study. For this reason, we deliberately restricted our interpretation to the most consistent findings, particularly those supported by previous evidence, while considering the remaining lipid alterations as exploratory observations that warrant validation in larger, independent cohorts. Nevertheless, our findings may provide a basis for identifying lipid predictors in CALD and support phenotype-specific lipid profiles for early patient stratification, particularly relevant now that X-ALD has been included in the newborn screening (Weinhofer et al., 2023; Honey et al., 2021).

It is also important to underline that the remodeling of LPC species may represent a possible common pathogenic feature in other lysosomal lipid storage disorders, beyond X-ALD, such recently reported by Mishra et al. (2024) for Niemann-Pick type C1 disease.

## Data Availability

The data presented in the study are deposited in the Zenodo repository, accession number 10.5281/zenodo.21374346.

## References

[B1] Azar C. Shor N. Nadjar Y. (2020). Adrenomyeloneuropathy masquerading as chronic myelitis. JAMA Neurol. 77, 522–523. 10.1001/jamaneurol.2020.0019 32091543

[B2] Berger J. Forss-Petter S. Eichler F. S. (2014). Pathophysiology of X-linked adrenoleukodystrophy. Biochimie 98, 135–142. 10.1016/j.biochi.2013.11.023 24316281 PMC3988840

[B3] Billington C. J. Rayannavar A. Tryon R. Kaye T. Gupta A. Lund T. C. (2025). Prognostication and biomarker potential of C26:0 lysophosphatidylcholine in adrenoleukodystrophy. JAMA Pediatr. 179, 465–467. 10.1001/jamapediatrics.2024.6774 39928311 PMC11811846

[B4] Bonaventura E. Alberti L. Lucchi S. Cappelletti L. Fazzone S. Cattaneo E. (2023). Newborn screening for X-linked adrenoleukodystrophy in Italy: diagnostic algorithm and disease monitoring. Front. Neurology 13, 1–11072256. 10.3389/fneur.2024.1376447 PMC9869129 36698902

[B45] Carneiro A. B. Iaciura B. M. F. Nohara L. L. Lopes C. D. Veas E. M. C. Mariano V. S. (2013). Lysophosphatidylcholine triggers TLR2- and TLR4-mediated signaling pathways but counteracts LPS-induced NO synthesis in peritoneal macrophages by inhibiting NF-κB translocation and MAPK/ERK phosphorylation. PLoS One 8 (9), e76233. 10.1371/journal.pone.0076233 24312681 PMC3848743

[B5] Di Rocco M. Doria-Lamba L. Caruso U. (2001). Monozygotic twins with X-linked adrenoleukodystrophy and different phenotypes. Ann. Neurol. 50, 424. 10.1002/ana.1220 11558805

[B46] Eichler F. S. Ren J. Q. Cossoy M. Rietsch A. M. Nagpal S. Moser A. B. (2008). Is microglial apoptosis an early pathogenic change in cerebral X-linked adrenoleukodystrophy? Ann. Neurol. 63 (6), 729–742. 10.1002/ana.21391 18571777

[B6] Eng L. Regelmann M. O. (2020). Adrenoleukodystrophy in the era of newborn screening. Curr. Opin. Endocrinol. Diabetes Obes. 27, 47–55. 10.1097/MED.0000000000000515 31789721

[B7] Engelen M. Kemp S. de Visser M. van Geel B. M. Wanders R. J. Aubourg P. (2012). X-linked adrenoleukodystrophy (X-ALD): clinical presentation and guidelines for diagnosis, follow-up and management. Orphanet J. Rare Dis. 13, 7–51. 10.1186/1750-1172-7-51 22889154 PMC3503704

[B8] Engelen M. Kemp S. Poll-The B. T. (2014). X-linked adrenoleukodystrophy: pathogenesis and treatment. Curr. Neurol. Neurosci. Rep. 14, 486. 10.1007/s11910-014-0486-0 25115486

[B9] Ferrer R. M. Jaspers Y. R. J. Dijkstra I. M. E. Breeuwsma N. van Klinken J. B. Romero C. (2025). Altered lipid profile and reduced neuronal support in human induced pluripotent stem cell-derived astrocytes from adrenoleukodystrophy patients. J. Inherit. Metab. Dis. 48, e12832. 10.1002/jimd.12832 39704488 PMC11660744

[B10] Fujitani N. Akashi T. Saito M. Morita M. So T. Oka K. (2024). Increased neurotoxicity of high-density lipoprotein secreted from murine reactive astrocytes deficient in a peroxisomal very-long-chain fatty acid transporter Abcd1. J. Inherit. Metab. Dis. 47, 289–301. 10.1002/jimd.12703 38146202

[B11] Gianni M. Goracci L. Schlaefli A. Di Veroli A. Kurosaki M. Guarrera L. (2022). Role of cardiolipins, mitochondria, and autophagy in the differentiation process activated by all-trans retinoic acid in acute promyelocytic leukemia. Cell Death Dis. 13, 30. 10.1038/s41419-021-04476-z 35013142 PMC8748438

[B12] Goracci L. Tortorella S. Tiberi P. Pellegrino R. M. Di Veroli A. Valeri A. (2017). Lipostar, a comprehensive platform-neutral cheminformatics tool for lipidomics. Anal. Chem. 89, 6257–6264. 10.1021/acs.analchem.7b01259 28471643

[B47] Hein S. Schönfeld P. Kahlert S. Reiser G. (2008). Toxic effects of X-linked adrenoleukodystrophy-associated, very long chain fatty acids on glial cells and neurons from rat hippocampus in culture. Hum. Mol. Genet. 17 (12), 1750–1761. 10.1093/hmg/ddn066 18344355

[B13] Honey M. I. J. Jaspers Y. R. J. Engelen M. Kemp S. Huffnagel I. C. (2021). Molecular biomarkers for adrenoleukodystrophy: an unmet need. Cells 10 (12), 3427. 10.3390/cells10123427 34943935 PMC8699919

[B14] Hubbard W. C. Moser A. B. Tortorelli S. Liu A. Jones D. Moser H. (2006). Combined liquid chromatography-tandem mass spectrometry as an analytical method for high throughput screening for X-linked adrenoleukodystrophy and other peroxisomal disorders: preliminary findings. Mol. Genet. Metabolism 89 (1–2), 185–187. 10.1016/j.ymgme.2006.05.001 16828324

[B15] Hubbard W. C. Moser A. B. Liu A. C. Jones R. O. Steinberg S. J. Lorey F. (2009). Newborn screening for X-linked adrenoleukodystrophy (X- ALD): validation of a combined liquid chromatography-tandem mass spectrometric (LC-MS/MS) method. Mol. Genet. Metabolism 97 (3), 212–220. 10.1016/j.ymgme.2009.03.010 19423374

[B16] Huffnagel I. C. van de Beek M.-C. Showers A. L. Orsini J. J. Klouwer F. C. C. Dijkstra I. M. E. (2017). Comparison of C26:0-carnitine and C26:0- lysophosphatidylcholine as diagnostic markers in dried blood spots from newborns and patients with adrenoleukodystrophy. Mol. Genet. Metabolism 122 (4), 209–215. 10.1016/j.ymgme.2017.10.012 29089175

[B17] Igarashi M. Schaumburg H. H. Powers J. Kishimoto Y. Koilodny E. Suzuki K. (1976). Fatty acid abnormality in adrenoleukodystrophy. J. Neurochem. 26, 851–860. 10.1111/j.1471-4159.1976.tb04462.x 965973

[B18] Jaspers Y. R. J. Ferdinandusse S. Dijkstra I. M. E. Barendsen R. W. van Lenthe H. Kulik W. (2020). Comparison of the diagnostic performance of C26:0- Lysophosphatidylcholine and very long-chain fatty acids analysis for peroxisomal disorders. Front. Cell Dev. Biol. 8, 690. 10.3389/fcell.2020.00690 32903870 PMC7438929

[B19] Jaspers Y. R. J. Dijkstra I. M. E. Engelen M. Kemp S. (2026). C26:0-lysophosphatidylcholine in X-linked adrenoleukodystrophy. Pharmacol. Ther. 285, 109070–109081. 10.1016/j.pharmthera.2026.109070 42320861

[B20] Jaspers R. J. Yska H. A. F. Bergner C. G. Dijkstra I. M. E. Huffnagel I. C. Voermans M. M. C. (2024). Lipidomic biomarkers in plasma correlate with disease severity in adrenoleukodystrophy. Commun. Med. (Lond). 10, 4–175. 10.1038/s43856-024-00605-9 PMC11387402 39256476

[B21] Jaspers Y. Meyer S. W. Pras-Raves M. L. Dijkstra I. M. E. Wever E. J. M. Dane A. D. (2024). Four-dimensional lipidomics profiling in X-linked adrenoleukodystrophy using trapped ion mobility mass spectrometry. J. Lipid Res. 65, 100567. 10.1016/j.jlr.2024.100567 38795862 PMC11234049

[B22] Kemp S. Valianpour F. Denis S. Ofman R. Sanders R. J. Mooyer P. (2005). Elongation of very long-chain fatty acids is enhanced in X-linked adrenoleukodystrophy. Mol. Genet. Metabolism 82, 144–151. 10.1016/j.ymgme.2004.09.015 15670720

[B23] Kloss A. Parisi L. Wang D. Hanus M. Golden R. Keefe K. (2026). Profiling of fatty acids and lipids in animal and human tissues yields new leads for disease progression biomarkers of X-linked adrenoleukodystrophy. J. Biol. Chem. 302, 112201. 10.1016/j.jbc.2026.112201 42036046 PMC13234235

[B24] Korenke G. C. Fuchs S. Krasemann E. Doerr H. G. Wilichowski E. Hunneman D. H. (1996). Cerebral adrenoleukodystrophy (ALD) in only one of monozygotic twins with an identical ALD genotype. Ann. Neurol. 40, 254–257. 10.1002/ana.410400221 8773611

[B48] Kruska N. Schönfeld P. Pujol A. Reiser G. (2015). Astrocytes and mitochondria from adrenoleukodystrophy protein (ABCD1)-deficient mice reveal that the adrenoleukodystrophy-associated very long-chain fatty acids target several cellular energy-dependent functions. Biochim. Biophys. Acta 1852 (5), 925–936. 10.1016/j.bbadis.2015.01.005 25583114

[B49] Launay N. Aguado C. Fourcade S. Ruiz M. Grau L. Riera J. (2015). Autophagy induction halts axonal degeneration in a mouse model of X-adrenoleukodystrophy. Acta Neuropathol. 129 (3), 399–415. 10.1007/s00401-014-1378-8 25549970 PMC4331612

[B50] Launay N. Ruiz M. Grau L. Ortega F. J. Ilieva E. V. Martínez J. J. (2017). Tauroursodeoxycholic bile acid arrests axonal degeneration by inhibiting the unfolded protein response in X-linked adrenoleukodystrophy. Acta Neuropathol. 133 (2), 283–301. 10.1007/s00401-016-1655-9 28004277 PMC5250669

[B25] Lee D. K. Long N. P. Jung J. Kim T. J. Na E. Kang Y. P. (2019). Integrative lipidomic and transcriptomic analysis of X-linked adrenoleukodystrophy reveals distinct lipidome signatures between adrenomyeloneuropathy and childhood cerebral adrenoleukodystrophy. Biochem. Biophys. Res. Commun. 508, 563–569. 10.1016/j.bbrc.2018.11.123 30509496

[B51] López-Erauskin J. Galino J. Bianchi P. Fourcade S. Andreu A. L. Ferrer I. (2012). Oxidative stress modulates mitochondrial failure and cyclophilin D function in X-linked adrenoleukodystrophy. Brain 135, 3584–3598. 10.1093/brain/aws292 23250880 PMC3525057

[B52] López-Erauskin J. Galino J. Ruiz M. Cuezva J. M. Fabregat I. Cacabelos D. (2013). Impaired mitochondrial oxidative phosphorylation in the peroxisomal disease X-linked adrenoleukodystrophy. Hum. Mol. Genet. 22 (16), 3296–3305. 10.1093/hmg/ddt186 23604518

[B44] Mallack E. J. Gao K. Engelen M. Kemp S. (2022). Structure and function of the ABCD1 variant database: 20 years, 940 pathogenic variants, and 3400 cases of adrenoleukodystrophy. Cells 11 (2), 283. 10.3390/cells11020283 35053399 PMC8773697

[B26] Marten L. M. Lüttgens M. S. Berečić B. Tiburcy M. Brüser C. Gupta S. (2026). Mitochondrial dysfunction and impaired oxidative stress defense as potential trigger of cerebral X-linked adrenoleukodystrophy. Free Radic. Biol. Med. 242, 654–666. 10.1016/j.freeradbiomed.2025.10.289 41177236

[B27] Mishra S. Kell P. Scherrer D. Dietzen D. J. Vite C. H. Berry-Kravis E. (2024). Accumulation of alkyl-lysophosphatidylcholines in Niemann-Pick disease type C1. J. Lipid Res. 65 (8), 100600. 10.1016/j.jlr.2024.100600 39048052 PMC11367646

[B28] Morales-Romero B. Gonz´alez de Aledo-Castillo J. M. Fern´andez Sierra C. Martínez Carreira C. Zaragoza Bonet C. Fern´andez Bonifacio R. (2024). Plasma C24:0- and C26:0-lysophosphatidylcholines are reliable biomarkers for the diagnosis of peroxisomal β-oxidation disorders. J. Lipid Res. 65 (3), 100516. 10.1016/j.jlr.2024.100516 38320654 PMC10910329

[B29] Pellegrino R. M. Di Veroli A. Valeri A. Goracci L. Cruciani G. (2014). LC/MS lipid profiling from human serum: a new method for global lipid extraction. Anal. Bioanal. Chem. 406, 7937–7948. 10.1007/s00216-014-8255-0 25381612

[B30] Petrillo S. D'Amico J. Nicita F. Torda C. Vasco G. Bertini E. S. (2022). Antioxidant response in Human X linked adrenoleukodystrophy fibroblasts. Antioxidants (Basel) 11, 2125. 10.3390/antiox11112125 36358497 PMC9686530

[B55] Prabutzki P. Schiller J. Engel K. M. (2024). Phospholipid-derived lysophospholipids in (patho)physiology. Atherosclerosis 398, 118569. 10.1016/j.atherosclerosis.2024.118569 39227208

[B56] Richmond P. A. van der Kloet F. Vaz F. M. Lin D. Uzozie A. Graham E. (2020). Multi-omic approach to identify phenotypic modifiers underlying cerebral demyelination in x-linked adrenoleukodystrophy. Front. Cell Dev. Biol. 8, 520. 10.3389/fcell.2020.00520 32671069 PMC7330173

[B31] Sahu R. K. Yang Y. Chung H. L. (2026). From lipid function to dysfunction: very long-chain fatty acids as emerging regulators of neuroinflammatory pathways. iScience 29, 114724. 10.1016/j.isci.2026.114724 41704776 PMC12907123

[B32] Salas Y. M. Kemper K. M. Shanmughapriya S. Langford D. Natarajaseenivasan K. (2026). Lipid metabolism and neurodegeneration: mechanistic insights and therapeutic targets. Ageing Res. Rev. 118, 103114. 10.1016/j.arr.2026.103114 41903861 PMC13276798

[B33] Tawbeh A. Gondcaille C. Saih F. E. Raas Q. Loichot D. Hamon Y. (2025). Impaired peroxisomal beta-oxidation in microglia triggers oxidative stress and impacts neurons and oligodendrocytes. Front. Mol. Neurosci. 18, 1542938. 10.3389/fnmol.2025.1542938 39958993 PMC11826809

[B34] Theda C. Moser A. B. Powers J. M. Moser H. W. (1992). Phospholipids in X-linked adrenoleukodystrophy white matter: fatty acid abnormalities before the onset of demyelination. J. Neurol. Sci. 110, 195–204. 10.1016/0022-510x(92)90028-j 1506859

[B35] Tiwari V. Simons M. (2025). Lipid metabolism and neuroinflammation: what is the link? J. Exp. Med. 222, e20241232. 10.1084/jem.20241232 40762805

[B36] Turk B. R. Theda C. Fatemi A. Moser A. B. (2020). X-linked adrenoleukodystrophy: pathology, pathophysiology, diagnostic testing, newborn screening and therapies. Int. J. Dev. Neurosci. 80, 52–72. 10.1002/jdn.10003 31909500 PMC7041623

[B37] Videbæk C. Melgaard L. Lund A. M. Grønborg S. W. (2023). Newborn screening for adrenoleukodystrophy: international experiences and challenges. Mol. Genet. Metab. 140, 107734. 10.1016/j.ymgme.2023.107734 37979237

[B38] Wang H. Davison M. D. Kramer M. L. Qiu W. Gladysheva T. Chiang R. M. S. (2022). Evaluation of neurofilament light chain as a biomarker of neurodegeneration in X-linked childhood cerebral adrenoleukodystrophy. Cells 11, 913. 10.3390/cells11050913 35269535 PMC8909395

[B39] Weinhofer I. Rommer P. Zierfuss B. Altmann P. Foiani M. Heslegrave A. (2021). Neurofilament light chain as a potential biomarker for monitoring neurodegeneration in X-linked adrenoleukodystrophy. Nat. Commun. 12, 1816. 10.1038/s41467-021-22114-2 33753741 PMC7985512

[B40] Weinhofer I. Rommer P. Gleiss A. Ponleitner M. Zierfuss B. Waidhofer-Söllner P. (2023). Biomarker-based risk prediction for the onset of neuroinflammation in X-linked adrenoleukodystrophy. eBioMedicine 96, 104781. 10.1016/j.ebiom.2023.104781 37683329 PMC10497986

[B41] Wilson R. Sargent J. R. (1993). Lipid and fatty acid composition of brain tissue from adrenoleukodystrophy patients. J. Neurochem. 61, 290–297. 10.1111/j.1471-4159.1993.tb03567.x 8515276

[B42] Yska H. A. F. Stellingwerff M. D. Roosendaal S. D. Voermans M. M. C. van der Knaap M. S. Pouwels P. J. W. (2025). Myelin-water imaging and multi-shell diffusion-weighted imaging in adults with adrenoleukodystrophy. Brain Commun. 7 (5), fcaf371. 10.1093/braincomms/fcaf371 41079752 PMC12514751

[B43] Yu J. Chen T. Guo X. Zafar M. I. Li H. Wang Z. (2022). The role of oxidative stress and inflammation in X-Link adrenoleukodystrophy. Front. Nutr. 9, 864358. 10.3389/fnut.2022.864358 35463999 PMC9024313

[B53] Zhu G. Zhou H. Ma Y. Liu R. Nie X. Qi W. (2025). A case of X-Linked adrenoleukodystrophy caused by a novel mutation with singular clinical manifestation: unilateral lower limb weakness. Neurol. Sci. 46 (1), 473–477. 10.1007/s10072-024-07828-7 39436520

